# Supplementary material for: Lymphocyte-specific protein 1 regulates mechanosensory oscillation of podosomes and actin isoform-based actomyosin symmetry breaking
Source: Nat Commun. 2018 Feb 6;9:515. doi: 10.1038/s41467-018-02904-x (PMC5802837; doi:10.1038/s41467-018-02904-x)
Supplement: Supplementary file 1 — Supplementary Information [file 41467_2018_2904_MOESM1_ESM.pdf]

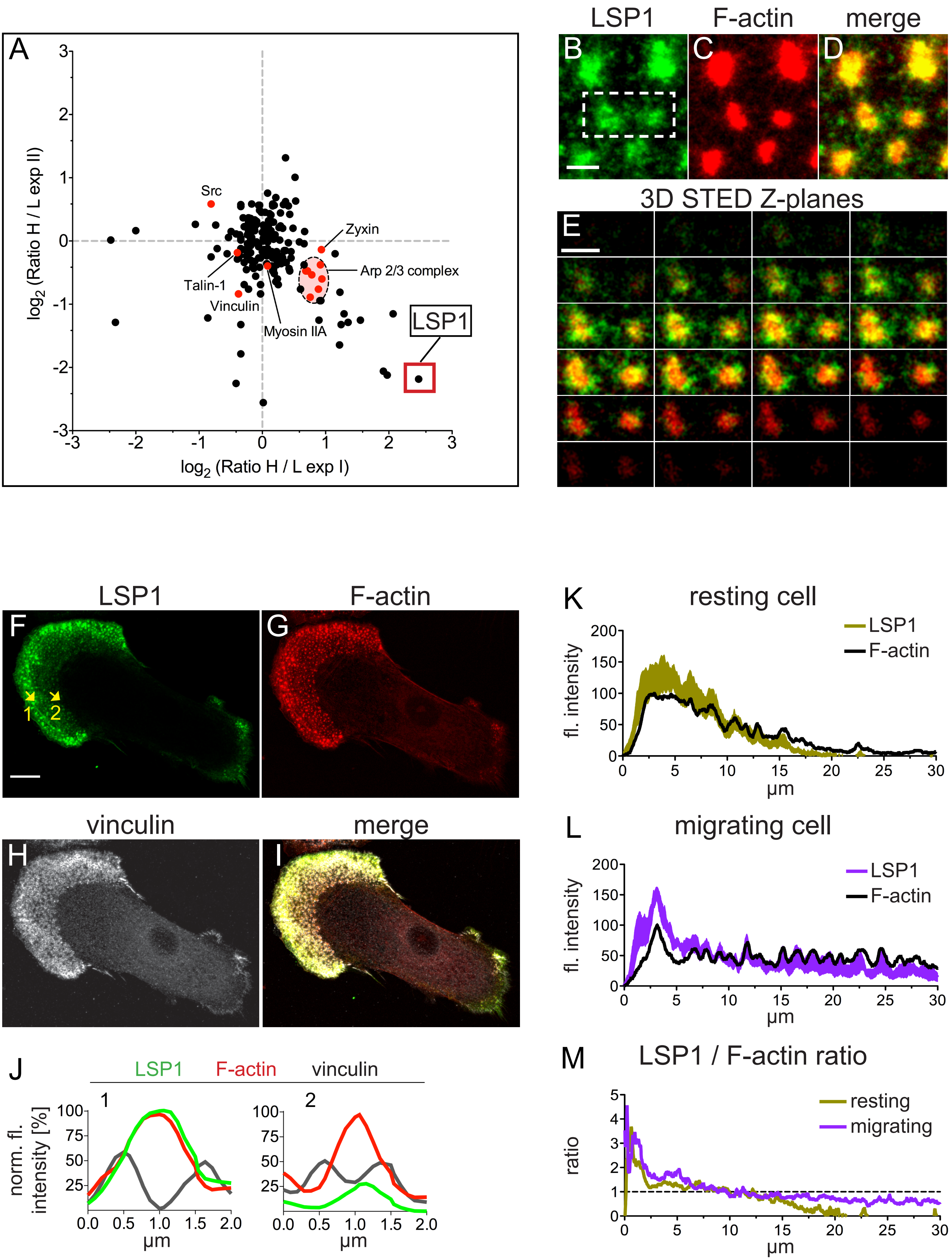

A
Src
Zyxin
Arp 2/3 complex
Talin-1
Vinculin
Myosin IIA
LSP1
log2 (Ratio H / L exp II)
log2 (Ratio H / L exp I)
LSP1
F-actin
merge
B
C
D
3D STED Z-planes
E
F
G
H
I
vinculin
merge
J
LSP1
F-actin
vinculin
norm. fl. intensity [%]
µm
K
resting cell
fl. intensity
L
migrating cell
M
LSP1 / F-actin ratio
resting
migrating
ratio

Supplementary Figure 1. SILAC analysis identifies LSP1 as a potential component of podosomes; STED analysis of podosomes; LSP1 is enriched at the leading edge of cells.

(A) Log2 ratios from SILAC analysis of podosome-enriched cell fractions from human macrophages. Proteins that are sensitive to inhibition of Src tyrosine signalling, and thus potentially podosome-associated, are enriched in the lower quadrant. Note respective enrichments of typical podosome components such as Arp2/3 complex, myosin IIA and vinculin, and highlighted position of LSP1. Modified, with permission, from (Cervero et al., 2012). (B-D) STED micrographs in extended focus mode of macrophage podosomes stained for LSP1 (B, green) and F-actin (C, red), with merge (D). Scale bars: 1 µm. Dashed box in (B) indicates detail region shown in several optical z planes from the apical to the ventral side in (E), which were used for 3D STED reconstruction shown in Fig. 1F. Note localization of LSP1 to the podosome cap and to podosome-connecting cables. Scale unit: 1 µm. (F-I) Confocal micrographs of a migratory macrophage stained for LSP1 using specific primary antibody and Alexa 488-labeled secondary antibody (F, green), for F-actin using Alexa 405-labeled phalloidin (G, red), and for vinculin using specific primary antibody and Alexa568-labeled secondary antibody (H, white), with merge (I). Scale bar: 10 µm. Yellow lines (1,2) in (F) indicate confocal planes used for measurements of respective fluorescence intensities shown in (J), with respective maximal fluorescence intensities set to 100%. Note enrichment of LSP1 at precursor podosome at the leading edge, compared to lower abundance at more internal successor podosome. (K-L) Analysis of LSP1 and F-actin intensities. Endogenous LSP1 and F-actin were stained with respective antibodies or Alexa 568-labeled phalloidin. Respective pixel intensities were measured along 30 µm starting with the cell edge, in both resting (K) and migratory (L) cells. Y-axis indicate relative fluorescence intensities, normalized to F-actin intensity values. (M) Respective LSP1/F-actin ratios based on graphs from (K,L). Note enrichment of LSP1 over F-actin in the cell periphery (5-10 µm from cell edges).

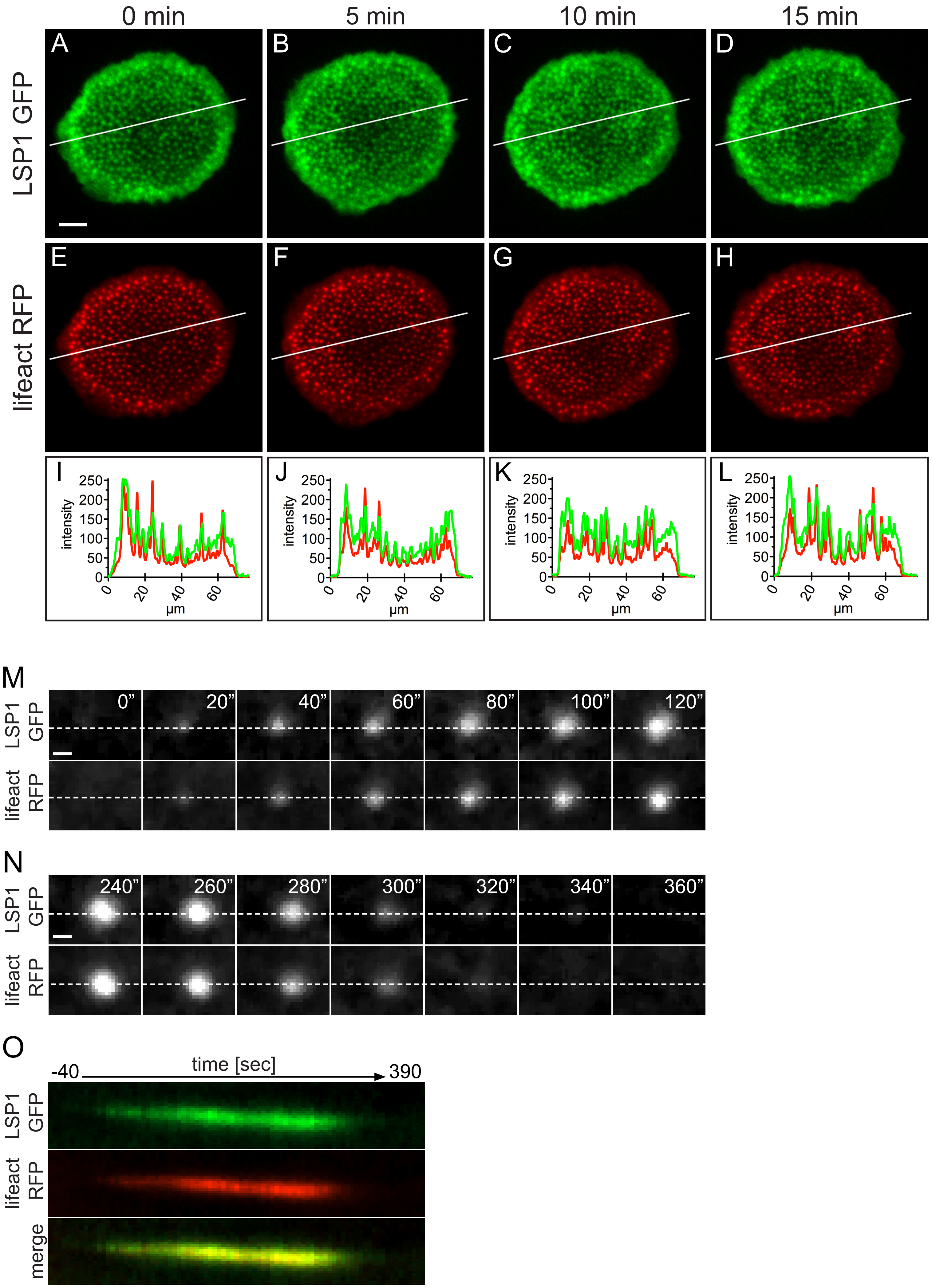

0 min
5 min
10 min
15 min
LSP1 GFP
lifeact RFP
A
B
C
D
E
F
G
H
I
J
K
L
intensity
µm
M
N
O
0”
20”
40”
60”
80”
100”
120”
240”
260”
280”
300”
320”
340”
360”
LSP1 GFP
lifeact RFP
time [sec]
-40
390
merge

Supplementary Figure 2. LSP1 and F-actin show comparable dynamics in quiescent macrophages.

(A-H) Still images from confocal live cell video of macrophage expressing LSP1-GFP (A-D, green) and lifeact-RFP (E-H) to label podosome cores. White lines in (A-H) indicate plane of pixels used for evaluation of fluorescence intensities shown in (I-L). Note comparable enrichment of LSP1-GFP at the periphery of quiescent cell, and also at individual podosomes Scale bar: 10 µm.
(M-O) LSP1-GFP and F-actin show similar dynamics during podosome lifetime. Still images from TIRF live cell video (time resolution of 5 sec/ frame) of LSP1-GFP (upper rows) and lifeact-RFP (lower rows) expressing macrophage showing de novo formation of single podosome (M) and dissolution of the same podosome (N). Respective fluorescence signals are shown in inverted greyscale. Scale bar: 1 µm. Time since start of experiment is indicated in sec. (O) Kymograph of podosome shown in (M,N), with time indicated in sec. Pixels used for generation of kymograph are indicated by dashed white line in (M-N).

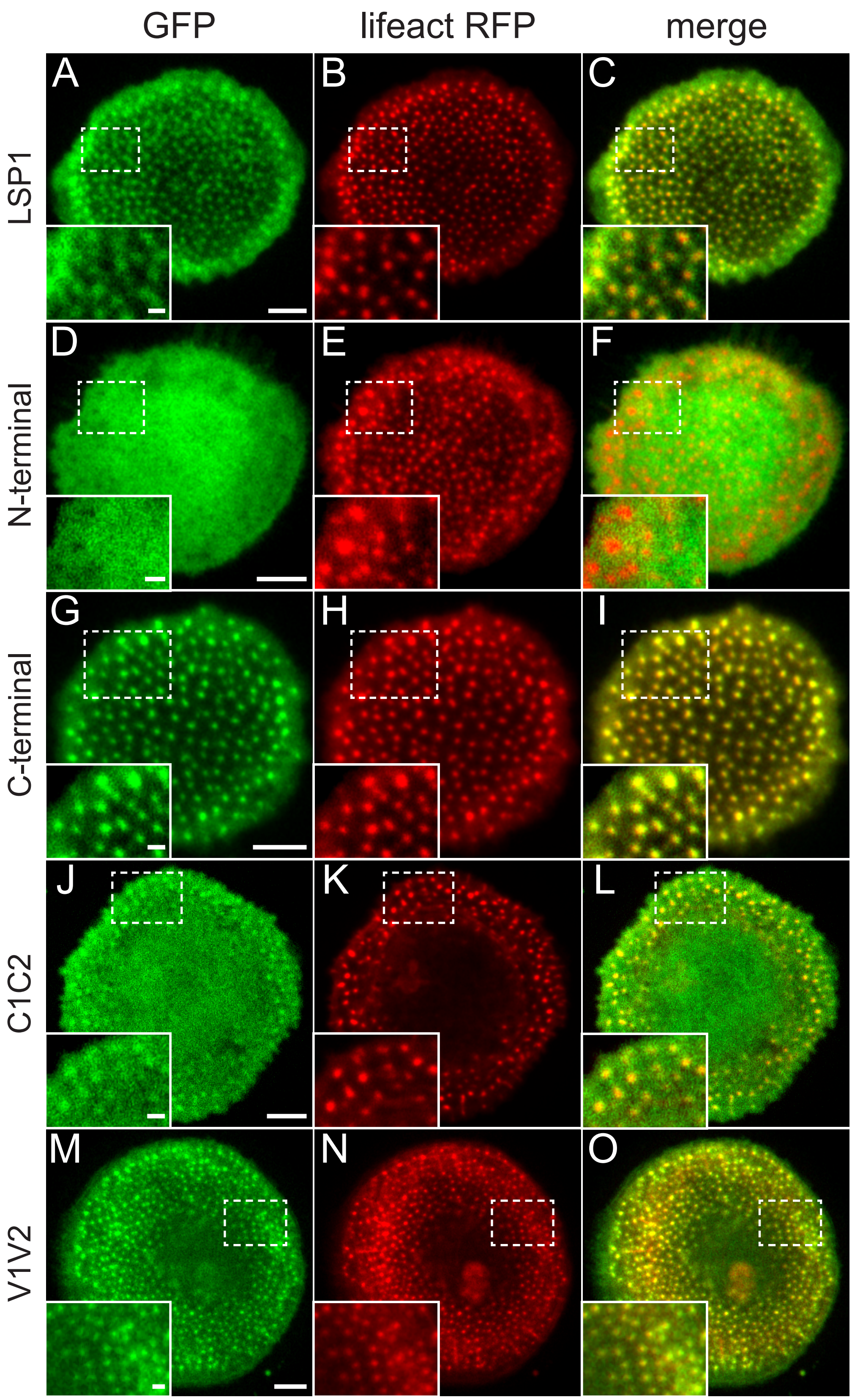
GFP
lifeact RFP
merge
LSP1
N-terminal
C-terminal
C1C2
V1V2
A
B
C
D
E
F
G
H
I
J
K
L
M
N
O

Supplementary Figure 3. The C-terminal villin-like domains of LSP1 are crucial for the localization to podosomes.

Confocal micrographs of macrophages expressing indicated constructs, with GFP signal in (A,D,G,J,M), and coexpressing lifeact-RFP to label podosome cores (B,E,H,K,N), with merges (C,F,I,L,O). Dashed boxes indicate detail regions shown as insets. Note unspecific localization of the N-terminal construct (D-F), but clear localization to podosomes of the C-terminal constructs, especially the V1/V2 containing ones (G-I,M-O). Scale bars: 10µm and 2µm for insets.

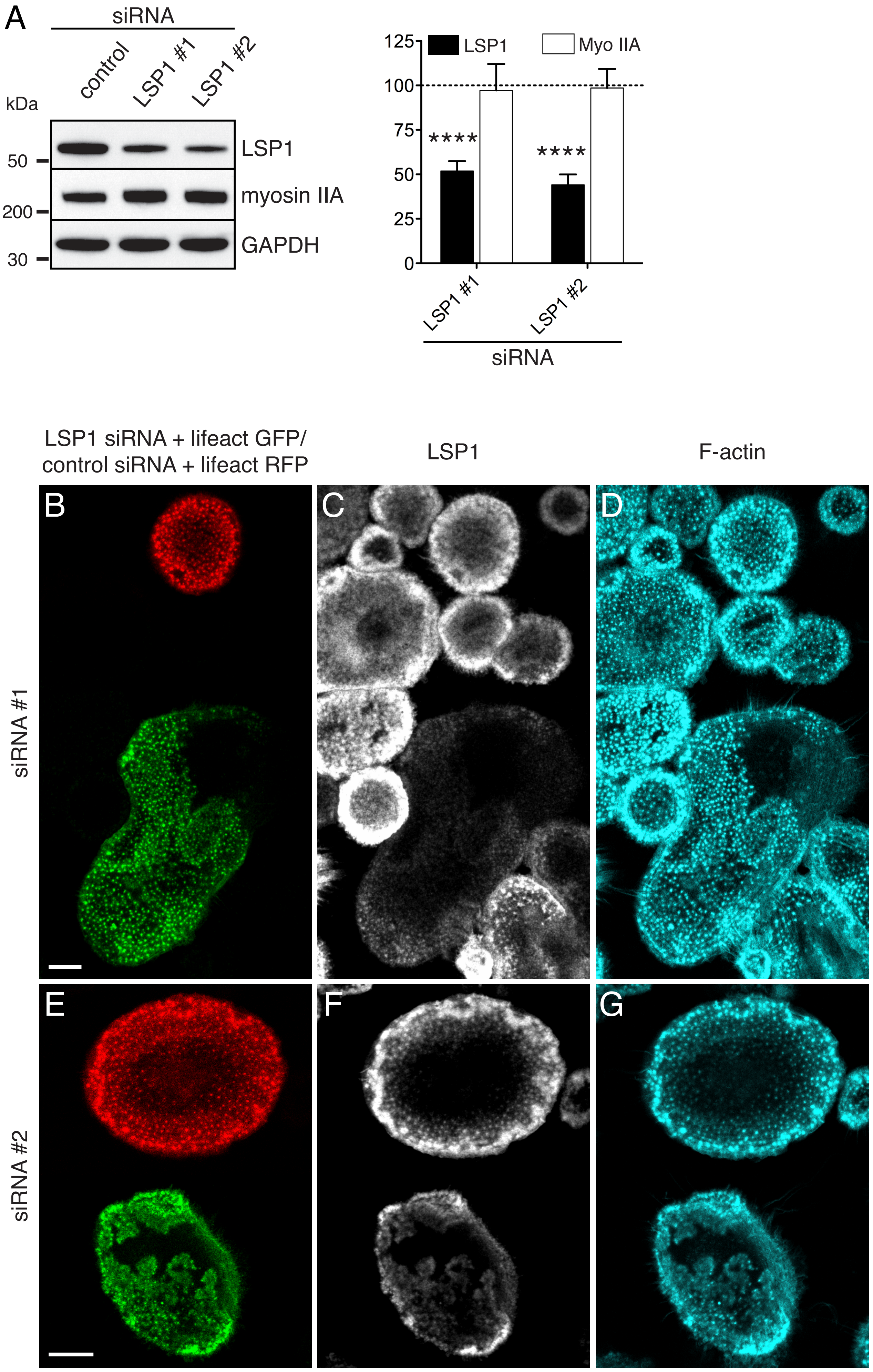
A
siRNA
control
LSP1 #1
LSP1 #2
kDa
50
200
30
LSP1
myosin IIA
GAPDH
LSP1
Myo IIA
125
100
75
50
25
0
****
****
LSP1 #1
LSP1 #2
siRNA
LSP1 siRNA + lifeact GFP/
control siRNA + lifeact RFP
LSP1
F-actin
B
C
D
siRNA #1
E
F
G
siRNA #2

Supplementary Figure 4. LSP1 knockdown does not change myosin IIA protein levels.

(A) Western blots from lysates of cells treated with either of two LSP1-specific siRNAs or luciferase siRNA as control, developed with anti-LSP1 or anti-myosin II antibodies and quantified as shown in the respective graph, with control set to 100%. Molecular weight indicated in kDa on left. Values are given as mean ± s.e.m. N=8, independent experiments; one-sample t-test; ****: $P<0.0001$. For specific values, see Suppl. Table S1. (B-G) Confocal micrographs of mixed macrophage populations. Cells treated with LSP1 siRNA are labeled by co-transfection and expression of lifeact-GFP (B,E; green), cells treated with control siRNA by co-transfection and expression of lifeact-RFP (B,E; red). Cells were stained for LSP1 using specific primary antibody (C,F; white) and Alexa 647-labeled secondary antibody, and for F-actin using Alexa 405-labeled phalloidin (D,G; blue). Scale bars: 10 µm.

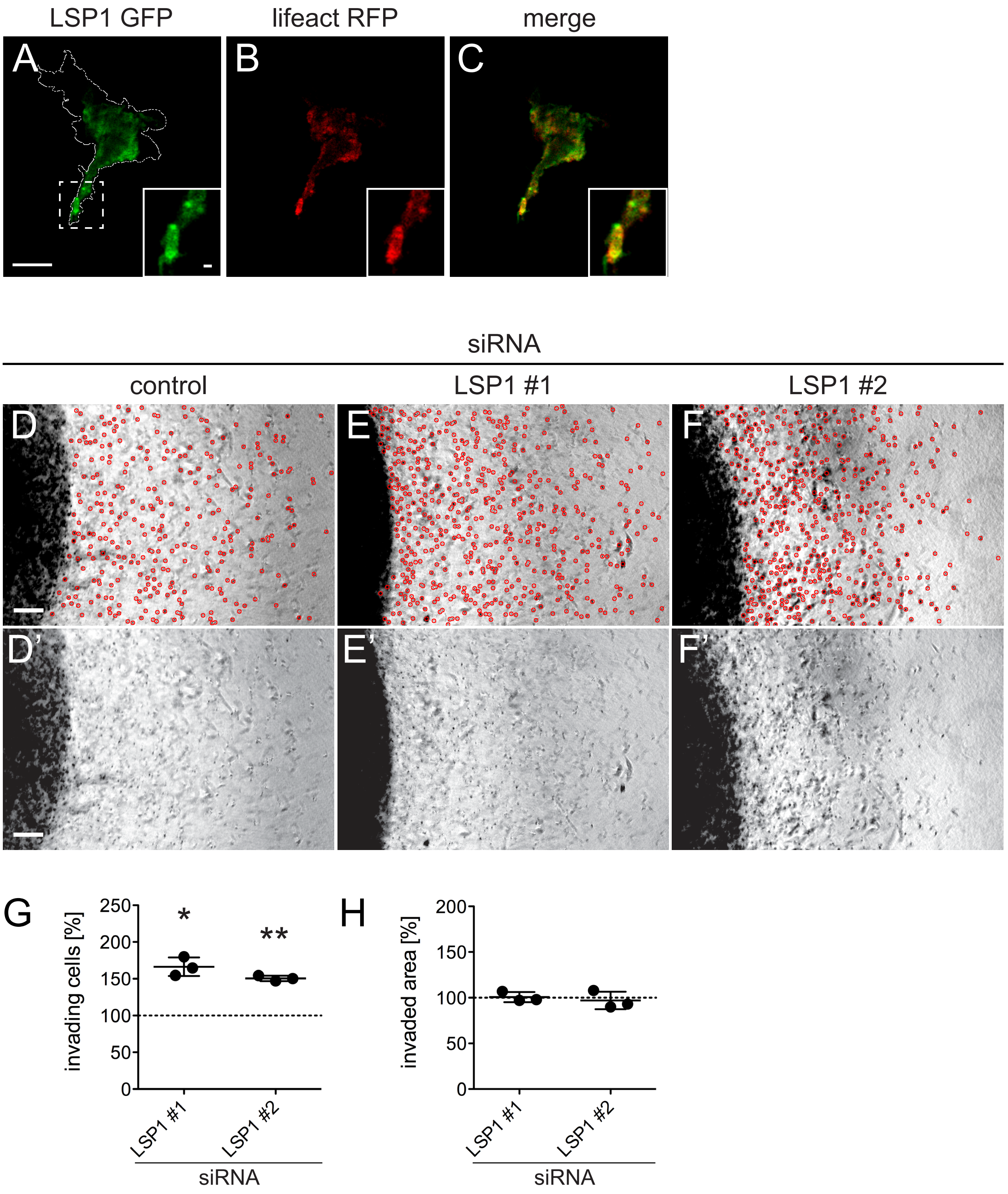

LSP1 GFP
lifeact RFP
merge
A
B
C
siRNA
control
LSP1 #1
LSP1 #2
D
E
F
D'
E'
F'
G
invading cells [%]
250
200
150
100
50
0
*
**
LSP1 #1
LSP1 #2
siRNA
H
invaded area [%]
200
150
100
50
0
LSP1 #1
LSP1 #2
siRNA

Supplementary Figure 5. LSP1 is a regulator of macrophage 3D invasion.

(A-C) LSP1 localizes to F-actin-rich protrusions in a 3D environment. Confocal micrograph of macrophage expressing LSP1-GFP (A) and lifeact-RFP (B), with merge (C), embedded in 3D collagen I matrix. Dashed line in (A) indicates cell outline in extended focus mode. Dashed box in (A) indicates detail region shown as insets. White bars: 10 µm and 1 µm in insets. (D-H) Evaluation of 3D macrophage invasion in a collagen plug invasion assay. (D-F) Brightfield micrographs of invading cells treated with indicated siRNA. Note dark zone of central plug with embedded macrophages, and bright zone of collagen matrix with invaded cells, visible as dark dots and highlighted by red circles. Scale bar: 100 µm. (D´-D´) Respective micrographs without red dot labelling. (G-H) Quantification of cell invasion into collagen matrix, at day 4 after seeding, with evaluation of cell numbers (G) and invaded area (H). Values for control siRNA were set to 100 %. Note enhanced number of invaded cells in case of LSP1 knockdown. Values are given as Mean ± S.D; 16 pictures per donor (N=3) were evaluated in each treatment; one-sample t-test; *: $P<0.05$, **: $P<0.01$. For specific values, see Suppl. Table S1

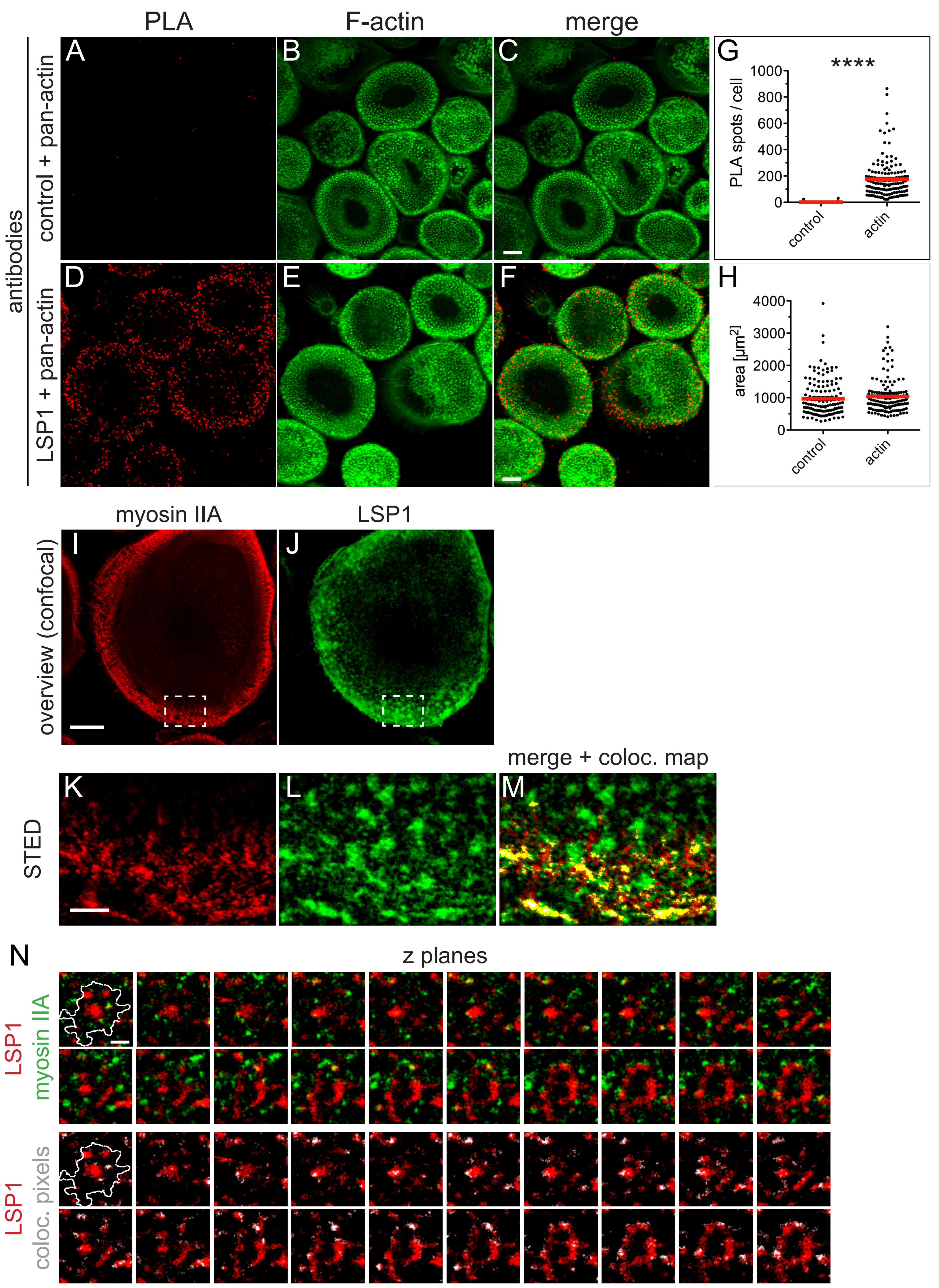

PLA
F-actin
merge
antibodies
control + pan-actin
LSP1 + pan-actin
A
B
C
D
E
F
G
****
PLA spots / cell
1000
800
600
400
200
0
control
actin
H
area [µm2]
4000
3000
2000
1000
0
control
actin
myosin IIA
LSP1
overview (confocal)
I
J
merge + coloc. map
STED
K
L
M
N
z planes
LSP1
myosin IIA
LSP1
coloc. pixels

Supplementary Figure 6. Subcellular interaction of LSP1 and actin.

(A-F) Confocal micrographs of macrophages subjected to a proximity ligation assay (PLA), using pan-actin-specific antibody, together with control IgG (A) or LSP1-specific antibody (D) and stained for F-actin (B,E), with merges (C,F). Scale bars: 10 µm. Note low background in (A) and PLA signals, especially in the cell periphery, in (D). (G,H) Statistical evaluation of number of PLA spots per cell (G) and cellular area analysed for respective PLAs (H). Each dot represents one cell. Data collected from 2 different donors. Values are given as mean ± s.e.m. ****: $P<0.0001$. For specific values, see Suppl. Table S1. (I-M) LSP1 and myosin IIA colocalize especially in the cell periphery of macrophages. (I,J) Confocal micrographs of macrophage stained for myosin IIA and LSP1 using specific antibodies. Scale bar: 10 µm. Dashed white boxes indicate detail region analysed by STED microscopy, as shown in (K,L), with merge of LSP1 signal (green), myosin IIA (red) and myosin IIA/LSP1 map of colocalizing pixels (yellow) (M). Scale bar: 2 µm. (N) Merges of STED micrographs of macrophage podosomes stained for LSP1 (red) and myosin IIA (green). Gallery shows optical z planes from apical to ventral side of the podosome. Dashed box in first image indicates circumference of a single podosome in extended focus mode. Lower rows: corresponding merges of LSP1 staining (red) and LSP1/myosin IIA colocalization pixels (white). Scale bar: 0.5 µm.

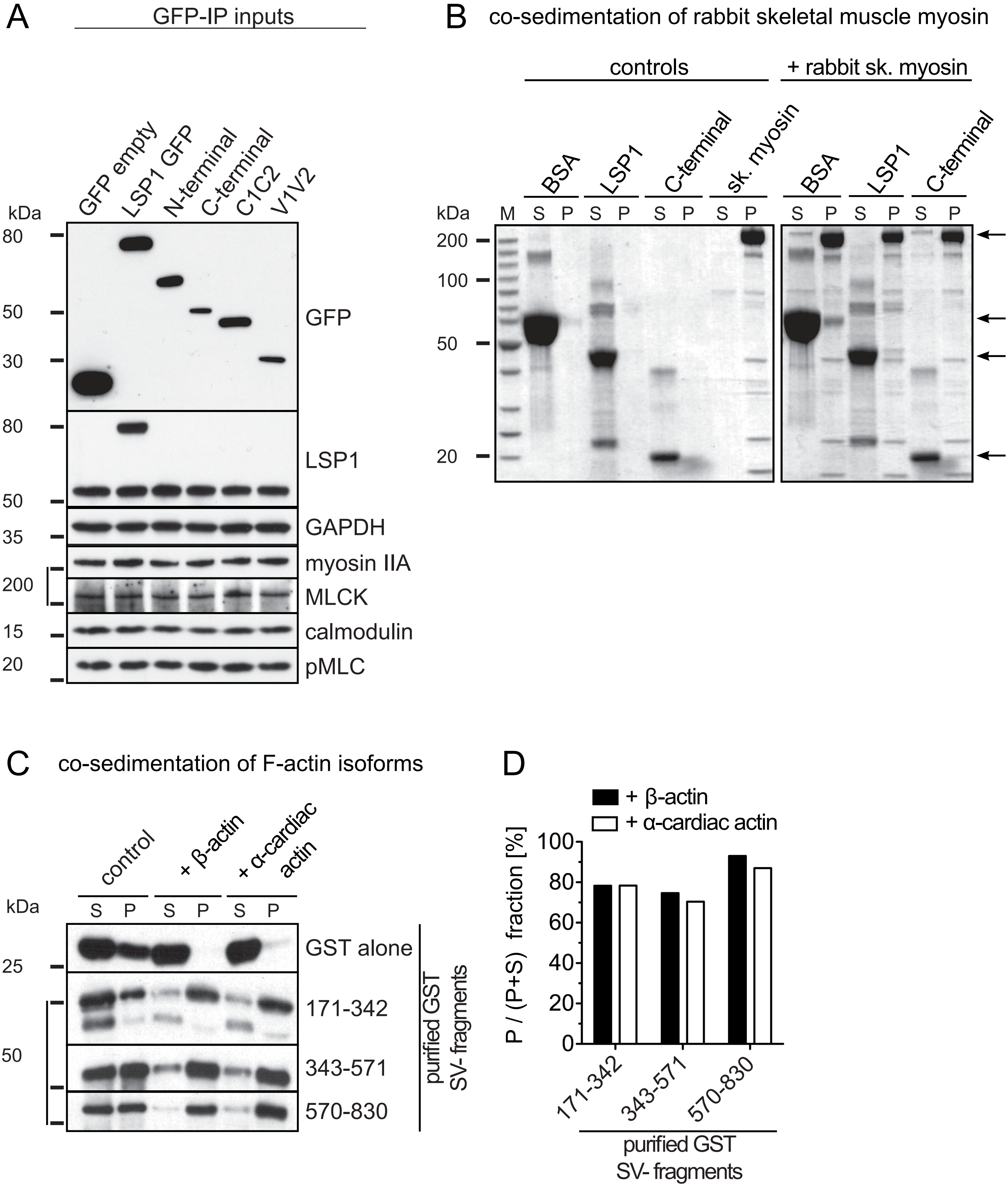

A
GFP-IP inputs
GFP empty
LSP1 GFP
N-terminal
C-terminal
C1C2
V1V2
kDa
80
50
30
GFP
80
50
LSP1
35
GAPDH
myosin IIA
200
MLCK
15
calmodulin
20
pMLC
B
co-sedimentation of rabbit skeletal muscle myosin
controls
+ rabbit sk. myosin
BSA
LSP1
C-terminal
sk. myosin
kDa
M
S
P
200
100
50
20
MHC
BSA
LSP1
LSP1 C-term
C
co-sedimentation of F-actin isoforms
control
+ β-actin
+ α-cardiac actin
kDa
25
50
GST alone
171-342
343-571
570-830
purified GST SV- fragments
D
+ β-actin
+ α-cardiac actin
P / (P+S) fraction [%]
0
20
40
60
80
100
171-342
343-571
570-830
purified GST SV- fragments

Supplementary Figure 7. Cosedimentation assays.

(A-B) LSP1 and myosin IIA binding is mediated by actin. (A). Inputs from immunoprecipitations shown in Fig. 5C. (B) Myosin cosedimentation assay. SDS PAGE gels from rabbit skeletal muscle myosin precipitation by ultracentrifugation in the presence of BSA, LSP1 full length or C-terminal constructs (right panel), with controls of individual proteins (left panel). Molecular weight is indicated in kDa. (C-D) Supervillin actin-binding regions cosediment to a comparable degree with β-actin and α-cardiac actin filaments. (C) Western blots from actin cosedimentation assays using pure β-actin or α-cardiac actin, in combination with GST as a control, or with GST fused constructs of the actin-binding regions of supervillin (SV171-342, SV343-571, SV570-830), as indicated. Lanes showing supernatant and pellet fractions are labelled with “S” and “P”, accordingly. Molecular weight is indicated in kDa. (D) Quantification of copelleted material as ratios of pelleted fraction versus input.

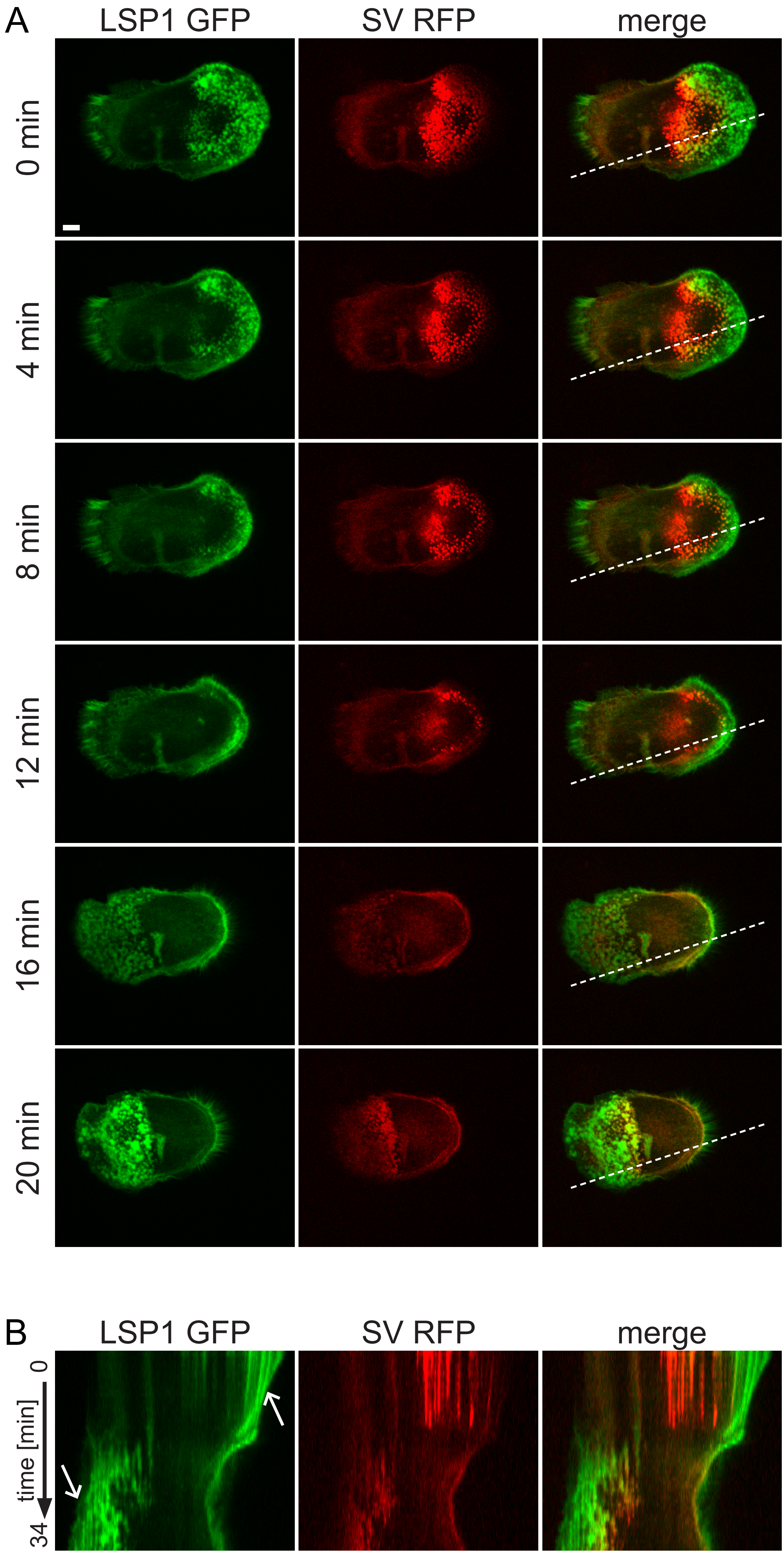

A
LSP1 GFP
SV RFP
merge
0 min
4 min
8 min
12 min
16 min
20 min
B
LSP1 GFP
SV RFP
merge
0
time [min]
34

Supplementary Figure 8. LSP1 and supervillin dynamically localize to different zones of migrating macrophages.

(A) Still images from confocal time lapse videos of macrophage coexpressing LSP1-GFP (green) and supervillin-RFP (red) with respective merges. White line in merges indicates pixels used to generate the respective kymograph shown in (B). Note localization of LSP1-GFP to the leading edge and leading edge-associated podosomes (precursors), whereas supervillin-RFP is mostly localized to more internally localized podosomes (successors). Note establishment of a new leading edge at timepoint 16 min, accompanied by formation of LSP1-GFP positive podosomes, with subsequent formation of a new zone of supervillin-RFP positive successor podosomes. Arrows in kymograph indicate LSP1-GFP positive leading edges. Time is indicated in min. Scale bar = 10 µm. See also Suppl. Video 5.

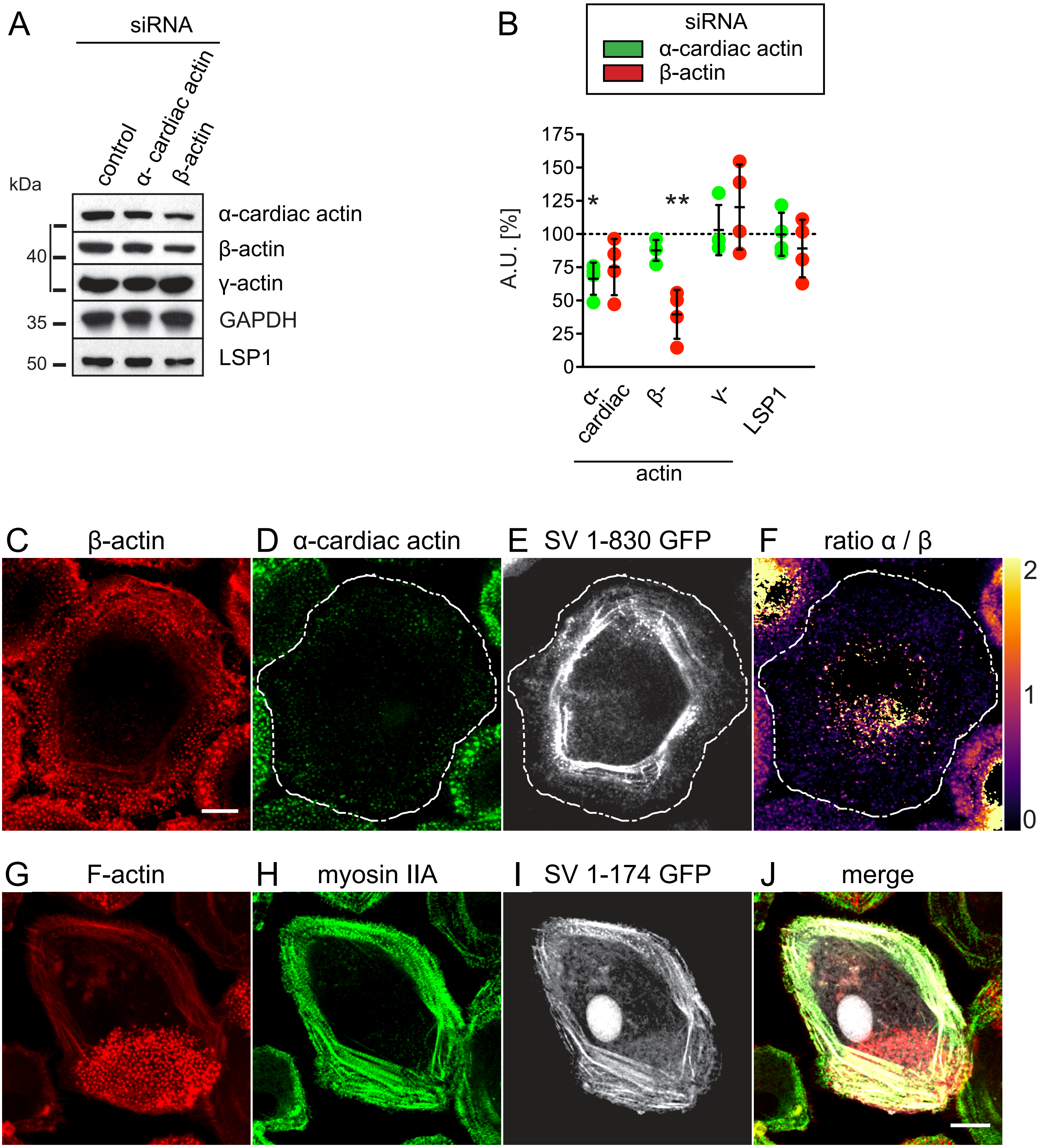
A
siRNA
control
α- cardiac actin
β-actin
kDa
40
35
50
α-cardiac actin
β-actin
γ-actin
GAPDH
LSP1
B
siRNA
α-cardiac actin
β-actin
A.U. [%]
175
150
125
100
75
50
25
0
*
**
α-cardiac
β-
γ-
LSP1
actin
C β-actin
D α-cardiac actin
E SV 1-830 GFP
F ratio α / β
2
1
0
G F-actin
H myosin IIA
I SV 1-174 GFP
J merge

Supplementary Figure 9. SiRNA-induced depletion of α-cardiac actin leads to relocalization of supervillin to the cell cortex.

(A-B) Western blot of lysates from macrophages treated with control siRNA, α-cardiac actin- or β-actin-specific siRNA, as indicated. Molecular weight is indicated in kDa. (B) Quantification of actin isoform and LSP1 levels in cells treated with indicated siRNAs. Values are given as Mean ± S.D; N=4; one-sample t-test; *: $P<0.05$, **: $P<0.01$. For specific values, see Suppl. Table S1. (C-F) Confocal micrographs of macrophages treated with α-cardiac actin specific siRNA and overexpressing SV1-830-GFP construct, stained for β-actin (C), α-cardiac actin (D), SV1-830 GFP signal (E), and ratio of α/β-actin, as indicated by colour scale (F). (G-J) Confocal micrographs of macrophages expressing the myosin II-binding region of supervillin (SV1-174-GFP), stained for F-actin (G), myosin IIA (H), SV1-174-GFP signal (I), with merge (J).
