# Supplementary material for: Lymphocyte-specific protein 1 regulates mechanosensory oscillation of podosomes and actin isoform-based actomyosin symmetry breaking
Source: Nat Commun. 2018 Feb 6;9:515. doi: 10.1038/s41467-018-02904-x (PMC5802837; doi:10.1038/s41467-018-02904-x)
Supplement: Supplementary file 3 — Description of Additional Supplementary Files [file 41467_2018_2904_MOESM3_ESM.pdf]

Description of Additional Supplementary Files

File Name: Supplementary Movie 1
Description: **3D STED animation.** 3D animated reconstruction of two podosomes from STED micrographs of macrophage stained for LSP1 (green) and F-actin (red), as shown in Fig. 1F.

File Name: Supplementary Movie 2
Description: **Podosome cluster dynamics, control cell.** Live imaging of macrophages targeted with control siRNA and expressing pLifeact-tagRFP (gray scale) to visualize F-actin, as shown in Fig. 2A. Experiment performed with Ultra*VIEW* VoX system (Perkin Elmer) spinning disk microscope, at 37° C and controlled atmosphere. Frames were acquired every 60 sec for 1h. Scale bar = 10 µm

File Name: Supplementary Movie 3
Description: **Podosome cluster dynamics, LSP1 knockdown cell (siRNA #1).** Live imaging of macrophage targeted with LSP1 siRNA #1 and expressing pLifeact-tagRFP (gray scale) to visualize F-actin, as shown in Fig. 2B. Experiment performed with Ultra*VIEW* VoX system (Perkin Elmer) spinning disk micrscope, at 37° C and controlled atmosphere. Frames were acquired every 60 sec for 1h. Scale bar = 10 µm

File Name: Supplementary Movie 4
Description: **Podosome cluster dynamics, LSP1 knockdown cell (siRNA #2).** Live imaging of macrophage targeted with LSP1 siRNA #2 and expressing pLifeact-tagRFP (gray scale) to visualize F-actin, as shown in Fig. 2C. Experiment performed with Ultra*VIEW* VoX system (Perkin Elmer) spinning disk microscope, at 37° C and controlled atmosphere. Frames were acquired every 60 sec for 1h. Scale bar = 10 µm

File Name: Supplementary Movie 5
Description: **Localization of LSP1 and supervillin in macrophage changing direction.** Live imaging of macrophage overexpressing GFP-LSP1 and supervillin-RFP, as shown in Suppl. Fig. 7A. Experiment performed with Ultra*VIEW* VoX system (Perkin Elmer) spinning disk microscope, at 37° C and controlled atmosphere. Frames were acquired every 35 sec. for 35 min. Scale bar = 10 µm

File Name: Supplementary Data 1
Description: **Values for podosome and cell analysis.**
