# Supplementary material for: Lymphocyte-specific protein 1 regulates mechanosensory oscillation of podosomes and actin isoform-based actomyosin symmetry breaking
Source: Nat Commun. 2018 Feb 6;9:515. doi: 10.1038/s41467-018-02904-x (PMC5802837; doi:10.1038/s41467-018-02904-x)
Supplement: Supplementary file 4 — Supplementary Data 1 [file 41467_2018_2904_MOESM4_ESM.pdf]

Supplementary Data 1. Values for podosome and cell analysis

**Fig. 2G**	**Mean velocity of podosome clusters [µm/min]**		
**Sample**	**Values**	**Mean ± SD**	
control siRNA	24	0.3099 ± 0.1841	
LSP1 siRNA #1	24	0.8719 ± 0.5838	
LSP1 siRNA #2	24	0.7312 ± 0.4697	
**One-way analysis of variance**			
P value			0.0001
P value summary			***
Are means signif. different? (P < 0.05)			Yes
Number of groups			3
F			10.34
R square			0.2307
**ANOVA Table**	**SS**	**df**	**MS**
Treatment (between columns)	4.105	2	2.053
Residual (within columns)	13.69	69	0.1984
Total	17.8	71	
**Bonferroni's Multiple Comparison Test**	**Mean Diff,**	**t**	**Summary**
control vs LSP1 #1	-0.562	4.37	****
control vs LSP1 #2	-0.4213	3.276	**

**Fig. 2H**	**Podosome lifetime [min]**		
**Sample**	**Values**	**Mean ± SD**	
control siRNA	24	9.921 ± 2.843	
LSP1 siRNA #1	24	6.560 ± 2.465	
LSP1 siRNA #2	24	6.554 ± 1.441	
**One-way analysis of variance**			
P value			< 0.0001
P value summary			****
Are means signif. different? (P < 0.05)			Yes
Number of groups			3
F			16.72
R square			0.3264
**ANOVA Table**	**SS**	**df**	**MS**
Treatment (between columns)	181	2	90.51
Residual (within columns)	373.5	69	5.413
Total	554.5	71	
**Bonferroni's Multiple Comparison Test**	**Mean Diff,**	**t**	**Summary**
control vs LSP1 #1	3.360	5.003	****
control vs LSP1 #2	3.367	5.013	****

Fig. 2M	Mean velocity of cells [µm/min]		
**Sample**	**Values**	**Mean ± SD**	
control siRNA	24	0.2380 ± 0.0977	
LSP1 siRNA #1	24	0.3329 ± 0.2569	
LSP1 siRNA #2	24	0.3521 ± 0.2486	
**One-way analysis of variance**			
P value			0.1488
P value summary			ns
Are means signif. different? (P < 0.05)			No
Number of groups			3
F			1.958
R square			0.05372
**ANOVA Table**	**SS**	**df**	**MS**
Treatment (between columns)	0.00004982	2	0.00002491
Residual (within columns)	0.0008776	69	0.00001272
Total	0.0009274	71	
**Bonferroni's Multiple Comparison Test**	**Mean Diff,**	**t**	**Summary**
control vs LSP1 #1	-0.001582	1.537	ns
control vs LSP1 #2	-0.001903	1.848	ns

Fig. 2N	Mean cell area [µm^2]		
**Sample**	**Values**	**Mean ± SD**	
control siRNA	24	1456 ± 407	
LSP1 siRNA #1	24	2226 ± 1025	
LSP1 siRNA #2	24	2771 ± 1506	
**One-way analysis of variance**			
P value			0.0003
P value summary			***
Are means signif. different? (P < 0.05)			Yes
Number of groups			3
F			9.020
R square			0.2073
**ANOVA Table**	**SS**	**df**	**MS**
Treatment (between columns)	20950000	2	10480000
Residual (within columns)	80140000	69	1161000
Total	101100000	71	
**Bonferroni's Multiple Comparison Test**	**Mean Diff,**	**t**	**Summary**
control vs LSP1 #1	-770.4	2.476	*
control vs LSP1 #2	-1315	4.227	***

Fig. 2O	Podosome distribution (clusters) [%]		
**Sample**	**Values**	**Mean ± SD**	
control siRNA	3	19.07 ± 7.067	
LSP1 siRNA #1	3	40.17 ± 6.250	
LSP1 siRNA #2	3	48.17 ± 5.244	
**One-way analysis of variance**			
P value			0.0032
P value summary			**
Are means signif. different? (P < 0.05)			Yes
Number of groups			3
F			17.46
R square			0.8534
**ANOVA Table**	**SS**	**df**	**MS**
Treatment (between columns)	1356	2	678.0
Residual (within columns)	233.0	6	38.84
Total	1589	8	
**Bonferroni's Multiple Comparison Test**	**Mean Diff,**	**t**	**Summary**
control vs LSP1 #1	-21.10	4.147	*
control vs LSP1 #2	-29.10	5.719	**

Fig. 2W	Cell shape analysis (AR ≥ 1.3 , circ. ≤ 0.8) [%]		
**Sample**	**Values**	**Mean ± SD**	
control siRNA	3	19.21 ± 7.285	
LSP1 siRNA #1	3	59.73 ± 9.941	
LSP1 siRNA #2	3	53.87 ± 8.454	
**One-way analysis of variance**			
P value			0.0084
P value summary			**
Are means signif. different? (P < 0.05)			Yes
Number of groups			3
F			11.78
R square			0.7971
**ANOVA Table**	**SS**	**df**	**MS**
Treatment (between columns)	2877	2	1439
Residual (within columns)	732.6	6	122.1
Total	3610	8	
**Bonferroni's Multiple Comparison Test**	**Mean Diff,**	**t**	**Summary**
control vs LSP1 #1	-40.52	4.491	**
control vs LSP1 #2	-34.66	3.842	*

Fig. 3E, 3L	Podosome oscillations [number of peaks]			
Sample	Values	Mean ± SD	Unpaired t-test	
			t	df
control GFP	30	2.900 ± 0.8030		
LSP1-GFP	30	4.333 ± 0.9942	6.143	58
control siRNA	30	3.133 ± 0.7761		
LSP1 siRNA #2	30	2.600 ± 1.133	2.128	58

Fig. 3F, 3M	Podosome oscillations [height of peaks]			
Sample	Values	Mean ± SD	Unpaired t-test	
			t	df
control GFP	87	20.46 ± 9.525		
LSP1-GFP	130	17.36 ± 11.77	2.046	215
control siRNA	93	21.42 ± 12.22		
LSP1 siRNA #2	78	32.78 ± 20.55	4.473	169

Fig. 3P	Podosome protrusion force [nN]			
Sample	Values	Mean ± SD	Unpaired t-test	
			t	df
control siRNA	21	2.451 ± 0.7480		
LSP1 siRNA #2	15	1.704 ± 0.8811	2.742	34

Fig. 4E	Myosin IIA fluorescence intensity at podosomes			
Sample	Values	Mean ± SD		
control siRNA	15	54.14 ± 21.31		
LSP1 siRNA #1	15	31.81 ± 16.25		
LSP1 siRNA #2	15	35.91 ± 11.93		
**One-way analysis of variance**				
P value				0.0018
P value summary				**
Are means signif. different? (P < 0.05)				Yes
Number of groups				3
F				7.394
R square				0.2604
**ANOVA Table**		**SS**	**df**	**MS**
Treatment (between columns)		4241	2	2121
Residual (within columns)		12045	42	286.8
Total		16286	44	
**Bonferroni's Multiple Comparison Test**		**Mean Diff,**	**t**	**Summary**
control vs LSP1 #1		22.34	3.612	**
control vs LSP1 #2		18.23	2.948	*

Fig. 4F	F-actin fluorescence intensity at podosomes	
**Sample**	**Values**	**Mean ± SD**
control siRNA	15	68.34 ± 22.16
LSP1 siRNA #1	15	65.01 ± 33.07
LSP1 siRNA #2	15	68.10 ± 37.43

One-way analysis of variance	
P value	0.9492
P value summary	ns
Are means signif. different? (P < 0.05)	No
Number of groups	3
F	0.05224
R square	0.002481

ANOVA Table	SS	df	MS
Treatment (between columns)	104.0	2	51.99
Residual (within columns)	41799	42	995.2
Total	41903	44	

Bonferroni's Multiple Comparison Test	Mean Diff,	t	Summary
control vs LSP1 #1	3.339	0.2898	ns
control vs LSP1 #2	0.2420	0.02101	ns

Fig. 4G	Myosin IIA ROI area [μm^2]	
**Sample**	**Values**	**Mean ± SD**
control siRNA	15	1.669 ± 0.5050
LSP1 siRNA #1	15	1.824 ± 0.7771
LSP1 siRNA #2	15	1.968 ± 0.5040

One-way analysis of variance	
P value	0.4118
P value summary	ns
Are means signif. different? (P < 0.05)	No
Number of groups	3
F	0.9063
R square	0.04137

ANOVA Table	SS	df	MS
Treatment (between columns)	0.6723	2	0.3362
Residual (within columns)	15.58	42	0.3709
Total	16.25	44	

Bonferroni's Multiple Comparison Test	Mean Diff,	t	Summary
control vs LSP1 #1	-0.1557	0.7001	ns
control vs LSP1 #2	-0.2993	1.346	ns

Fig. 4H	F-actin ROI area [μm^2]				
**Sample**	**Values**	**Mean ± SD**			
control siRNA	15	0.4534 ± 0.2088			
LSP1 siRNA #1	15	0.4103 ± 0.1533			
LSP1 siRNA #2	15	0.5610 ± 0.1795			
**One-way analysis of variance**					
P value					0.0766
P value summary					ns
Are means signif. different? (P < 0.05)					No
Number of groups					3
F					2.733
R square					0.1151
**ANOVA Table**			**SS**	**df**	**MS**
Treatment (between columns)			0.1810	2	0.09048
Residual (within columns)			1.391	42	0.03311
Total			1.572	44	
**Bonferroni's Multiple Comparison Test**			**Mean Diff,**	**t**	**Summary**
control vs LSP1 #1			-0.04311	0.6488	ns
control vs LSP1 #2			-0.1077	1.621	ns

Fig. 4L	PLA spots / cell			
**Sample**	**Values**	**Mean ± SD**	**Unpaired t-test**	
			**t**	**df**
IgG + myo IIA	130	2.43 ± 4.76		
LSP1 + myo IIA	130	72.98 ± 88.84	9.04	258

Fig. 7E	Correlation and linear regression of actin isoforms	
**Sample**	**Pearson r**	**R^2**
α-cardiac actin	0.3934	0.1548
β-actin	- 0.8055	0.6488
γ-actin	- 0.6515	0.4244

Fig. 8B	F-actin co-sedimentation [P / (S+P)] [%]			
**Sample**	**Values**	**Mean ± SD**	**Unpaired t-test**	
			**t**	**df**
LSP1 + β-actin	5	84.00 ± 6.015		
LSP1 + α-cardiac actin	5	44.58 ± 4.465	11.77	8

Fig. 9M	Mean cell area [µm²]		
**Sample**	**Values**	**Mean ± SD**	
control siRNA	120	1552 ± 747.1	
LSP1 siRNA #1	120	1548 ± 728.0	
LSP1 siRNA #2	120	1794 ± 1000	
**One-way analysis of variance**			
P value			0.0341
P value summary			*
Are means signif. different? (P < 0.05)			Yes
Number of groups			3
F			3.411
R square			0.01880
**ANOVA Table**	**SS**	**df**	**MS**
Treatment (between columns)	4743000	2	2372000
Residual (within columns)	247600000	356	695377
Total	252300000	358	
**Bonferroni's Multiple Comparison Test**	**Mean Diff,**	**t**	**Summary**
control vs LSP1 #1	4.116	0.03823	ns
control vs LSP1 #2	-242.1	2.244	ns

Fig. 9N	Podosome distribution (polarised) [%]		
**Sample**	**Values**	**Mean ± SD**	
control siRNA	3	45.70 ± 3.940	
LSP1 siRNA #1	3	27.30 ± 7.467	
LSP1 siRNA #2	3	24.00 ± 6.065	
**One-way analysis of variance**			
P value			0.0091
P value summary			**
Are means signif. different? (P < 0.05)			Yes
Number of groups			3
F			11.39
R square			0.7915
**ANOVA Table**	**SS**	**df**	**MS**
Treatment (between columns)	820.3	2	410.2
Residual (within columns)	216.1	6	36.02
Total	1036	8	
**Bonferroni's Multiple Comparison Test**	**Mean Diff,**	**t**	**Summary**
control vs LSP1 #1	18.40	3.755	*
control vs LSP1 #2	21.70	4.428	**

Fig. 9U	Cell shape analysis (AR ≥ 1.3 , circ. ≤ 0.8) [%]		
**Sample**	**Values**	**Mean ± SD**	
control siRNA	3	27.74 ± 9.415	
LSP1 siRNA #1	3	9.963 ± 6.314	
LSP1 siRNA #2	3	16.98 ± 5.114	
**One-way analysis of variance**			
P value			0.06
P value summary			ns
Are means signif. different? (P < 0.05)			No
Number of groups			3
F			4.662
R square			0.6085
**ANOVA Table**	**SS**	**df**	**MS**
Treatment (between columns)	480.8	2	240.4
Residual (within columns)	309.3	6	51.56
Total	790.1	8	
**Bonferroni's Multiple Comparison Test**	**Mean Diff,**	**t**	**Summary**
control vs LSP1 #1	17.77	3.031	*
control vs LSP1 #2	10.75	1.834	ns

Suppl. Fig. 4A	Western blot quantification of LSP1 knockdown [% vs control]				
**Sample**	**Values**	**Mean ± SD**	**One sample t-test (theor.mean = 100)**		
			**t**	**df**	**P value**
LSP1 #1	8	51.89 ± 15.78	8.622	7	< 0.0001
LSP1 #2	8	44.17 ± 16.30	9.687	7	< 0.0001

Suppl. Fig. 5G	Mean number of cells invading into 3D collagen I [% vs control]				
**Sample**	**Values**	**Mean ± SD**	**One sample t-test (theor.mean = 100)**		
			**t**	**df**	**P value**
LSP1 siRNA #1	3	166.00 ± 12.73	9.030	2	0.0120
LSP1 siRNA #2	3	150.60 ± 3.69	23.75	2	0.0018

Suppl. Fig. 5H	Mean value of invaded area [% vs control]				
**Sample**	**Values**	**Mean ± SD**	**One sample t-test (theor.mean = 100)**		
			**t**	**df**	**P value**
LSP1 siRNA #1	3	100.70 ± 5.508	0.2097	2	0.8534
LSP1 siRNA #2	3	97.00 ± 9.644	0.5388	2	0.6440

Suppl. Fig. 6G	PLA spots / cell			
Sample	Values	Mean ± SD	Unpaired t-test	
			t	df
IgG + pan-actin	140	1.12 ± 3.37		
LSP1 + pan-actin	140	175.90 ± 148.50	13.92	278

Suppl. Fig. 9B	Western blot quantification of α-cardiac actin knockdown [% vs control]				
Sample	Values	Mean ± SD	One sample t-test (theor.mean = 100)		
			t	df	P value
α-card act. /GAPDH	4	66.44 ± 12.16	5.522	3	0.0117
β-act. /GAPDH	4	87.68 ± 7.881	3.128	3	0.0522
γ-act. /GAPDH	4	103.0 ± 18.84	0.3159	3	0.7728
LSP1 /GAPDH	4	99.84 ± 16.15	0.02010	3	0.9852

Suppl. Fig. 9B	Western blot quantification of β-actin knockdown [% vs control]				
Sample	Values	Mean ± SD	One sample t-test (theor.mean = 100)		
			t	df	P value
α-card act. /GAPDH	4	75.19 ± 21.15	2.346	3	0.1006
β-act. /GAPDH	4	39.58 ± 18.36	6.580	3	0.0071
γ-act. /GAPDH	4	120.3 ± 31.99	1.270	3	0.2937
LSP1 /GAPDH	4	89.14 ± 21.67	1.002	3	0.3901
